# Prognostic utility of serum NT-proBNP (fragments 1-76aa and 13-71aa) and galectin-3 in predicting death and re-hospitalisation due to cardiovascular events in patients with heart failure

**DOI:** 10.1007/s00380-023-02296-z

**Published:** 2023-08-03

**Authors:** Xi Zhang, Yunxia Wan, Nuwan Karunathilaka, Wandy Chan, Karam Kostner, Gunter Hartel, Andrew J. S. Coats, John J. Atherton, Chamindie Punyadeera

**Affiliations:** 1 Saliva and Liquid Biopsy Translational Laboratory, Griffith Institute for Drug Discovery, Griffith University, 46, Don Young Rd, Nathan, QLD 4111 Australia; 2 Saliva and Liquid Biopsy Translational Laboratory, The School of Biomedical Sciences, Institute of Health and Biomedical Innovation, Queensland University of Technology, Brisbane, QLD Australia; 3 The Prince Charles Hospital, Brisbane, QLD Australia; 4 Department of Cardiology, Mater Adult Hospital, Brisbane, QLD Australia; 5 Statistics Unit, QIMR Berghofer Medical Research Institute, Brisbane, QLD Australia; 6 Faculty of Medicine, Monash University, Melbourne, VIC Australia; 7 University of Warwick, Coventry, UK; 8 Cardiology Department, Faculty of Medicine, Royal Brisbane and Women’s Hospital and University of Queensland, Brisbane, QLD Australia; 9 Translational Research Institute, Woolloongabba, Australia; 10 Menzies Health Institute Queensland (MHIQ), Griffith University, Gold Coast, QLD Australia; 11 School of Medicine, University of Queensland, Brisbane, Australia; 12 Monash University, Clayton, Australia

**Keywords:** Heart failure, Prognosis, Gal-3, NT-proBNP

## Abstract

Patients with heart failure (HF) are at a higher risk of rehospitalisation. In this study, we investigated the prognostic utility of galectin-3 (Gal-3) and NT-proBNP fragments (1-76aa and 13-71aa) as biomarkers to predict outcomes for patients with HF. We collected blood samples from patients with HF (*n* = 101). Gal-3 and NT-proBNP fragments (1–76aa and 13–71aa) concentrations were measured by immunoassay. Survival analysis and Cox proportional regression models were used to determine the prognostic utility of Gal-3 and NT-proBNP fragments. In patients with increased baseline levels of NT-proBNP_1-76_ the time to primary endpoint (cardiovascular death or re-hospitalisation) was significantly shorter (*p* = 0.0058), but not in patient with increased baseline levels of Gal-3 or NTproBNP_13-71_. Patients with increased levels of NT-proBNP_13-71aa_ at 1 month showed reduced time to the primary endpoint (*p* = 0.0123). Our findings demonstrated that Gal-3 and NT-proBNP can be used as prognostic biomarkers to stratify patients with HF.

**Supplementary Information:**

The online version contains supplementary material available at 10.1007/s00380-023-02296-z.

## Introduction

Heart failure (HF) is a major global health problem, affecting more than 64.3 million people worldwide [1]. Patients with HF have a relatively higher risk of repeated hospitalisations, which is associated with a considerable loss of quality of life and accounts for most of the healthcare costs associated with HF. Nearly a quarter of patients with HF over the age of 65 years are re-hospitalised within a month of being discharged [2]. Whilst there are clear benefits of HF management services, these services are resource-intensive, and are not universally available, especially to those patients from rural and remote communities where the disease burden is high. Hence outcomes remain poorer and 20% of patients with HF dye within 1 year of hospitalisation [3]. One of the reasons for adverse outcomes and reliance on repeated hospitalisations is the lack of accurate biomarkers to stratify the risk in patients with HF.

Prognostic biomarkers for HF play a crucial role in predicting disease outcomes and aiding in patient management strategies. Cardiac troponins (Troponin T and I) signify myocardial injury and are particularly relevant in acute decompensated heart failure [4]. ST2, a marker of cardiac stretch or stress, provide further insight into heart damage at a minute level [5]. Additionally, C-reactive protein (CRP) serves as a general indicator of body-wide inflammation which can exacerbate heart failure [6]. Cystatin C is a vital marker of renal function, its deterioration often accompanies advanced heart failure. Furthermore, adrenomedullin (ADM) and Mid-regional pro-adrenomedullin (MR-proADM), linked with vasodilation and myocardial structure alteration, provide valuable information regarding the physiological status of the heart [7]. Some of these biomarkers can be elevated in conditions other than heart failure, such as kidney disease, lung diseases, sepsis, and even intense physical exercise. For a precise prognosis prediction in patients with HF, there is a need for more specific biomarkers. Galectin-3 (Gal-3) causes progressive fibrosis, and probably plays a mechanistic role in the inexorable progression of HF [8]. The measurement of Gal-3 levels in blood is part of routine clinical practice in some parts of the world and has shown to be clinically useful in identifying patients at an increased risk of undergoing adverse outcomes [9]. Repeated measurements of Gal-3 levels have demonstrated superior clinical utility than single measurements of serum Gal-3 [10, 11]. In the coordinating study evaluating outcomes of advising and counselling in HF, higher levels of serum Gal-3 levels were associated with a greater risk of death or hospitalisation due to HF [10]. Similarly, the Deventer- Alkmaar Heart Failure study [11] evaluated 232 patients with HF and found that serum Gal-3 was a significant predictor of mortality (follow-up up to 6.5 years) even after adjusting for other variables. However, a limitation of the Deventer study was that the blood sampling was carried out at hospital discharge and 6 months, by which time 40–50% of patients would have either died or been rehospitalised, limiting its clinical utility.

Salah et al*.* found that NT-proBNP is also predictive of all-cause mortality and HF hospitalisations in patients with HF with preserved and reduced ejection fractions [12]. However, most studies have only measured the blood biomarkers either at a single time point or multiple time points which may miss the opportunity for intervention (after many events have happened). While these studies reported significant prognostic value of the biomarkers, the performance characteristics of these biomarkers were found to be suboptimal (overall accuracy ranged from 61% to 72%) [11, 13]. Studies have also shown that circulating NT-proBNP has truncated forms of endogenous NT-proBNP and are glycosylated which may cause inaccuracies and assay-dependent variations in the measurement [14, 15]. Studies have also shown variability in results when using the full-length NT-proBNP fragment either as a diagnostic or prognostic biomarker [16].

We hypothesise that quantifying Gal-3 and NT-proBNP (NT-proBNP_1-76aa_ and NT-proBNP_13-71aa_) concentrations in blood samples collected from HF patients can identify those patients at a higher risk of all-cause mortality and/or re-hospitalisations. The aims of this study are three-folds: firstly to develop an assay that can accurately measure NT-proBNP 13-71aa levels in serum samples from patients with HF; secondly, to investigate whether frequent measurements of Gal-3 and or NT-proBNP concentrations following hospitalisation improves risk stratification; and finally to develop a Gal-3 and NT-proBNP driven stratification method (in addition to clinical predictors) to identify patients with HF at a higher risk of all-cause mortality and/or rehospitalisation.

## Material and methods

### Study design and participants

This is a longitudinal, propective follow-up study with initial sampling at hospital discharge (baseline, time 1) and repeat sampling during subsequent clinical visits at 1 month (time 2) to establish the prognostic utility of repeated sampling of NT-proBNP_1-76aa_, NT-proBNP_13-71aa_ and Gal-3 to predict cardiovascular death or rehospitalisation (primary outcomes) for up to 4.5 years. We selected 1-month post hospital discharge as a time point because 1 in 4 HF patients are readmitted to hospital within 1 month [17–20]. Clinical information at baseline was collected during hospital stay and subsequent clinical visits. The follow-up clinical outcomes were obtained through hospital records and by contacting the patients’ general practitioner.

### Participants

This study complies with the 2013 Declaration of Helsinki [21] and the Australian Code for Responsible Conduct of Research [22]. Research ethics approval for sample collection was obtained from the Human Research Ethics Committee of the Mater Adult Hospital (approval number: HREC/13/MHS/142 (1806QA). Informed consent was obtained from all participants.

HF was determined by the treating cardiologist in accordance with the most recent clinical guidelines [23]. Patients who were hospitalised for acute decompensated HF and those who met the inclusion criteria was approached by the clinical coordinator to consent for the study. We did not keep a screening log of the patients, as this was an observational study. The inclusion criteria for the patients included the following: a left ventricular ejection fraction (LVEF, determined by echocardiogram) ≤ 40%, age between 18 and 100 years, willingness to participate for the duration of the trial period, and provision of written informed consent to participate in our study. Patients were recruited from the cardiology departments from the Mater Adult Hospital, the Prince Charles Hospital and the Royal Brisbane and Women’s Hospitals, in Brisbane, Australia. HF severity in these patients was determined using the New York Heart Association (NYHA) Functional Class based on clinical symptoms [24] identified by the treating cardiologists. The parameters collected are summarised in supplementary Table 1. The primary clinical endpoint was defined as cardiovascular death or cardiovascular hospitalisation within 4.5 years of collection of the samples (see Supplementary Table 1). The clinical endpoint of each patient was adjudicated by the cardiologists blinded to the biomarker measurement data using the patients’ clinical records. The follow-up ceased once patients experienced their first primary endpoint event or experienced no event after 4.5 years. We lost patients due to Covid-19 restriction to accessing hospitals.

### Galectin-3 ELISA assay

To determine the Gal-3 concentrations in the serum samples, we purchased the R&D® human Galectin-3 Duoset ELISA kit (cat#: DY1154) (R&D Systems, Minneapolis, Minnesota, United State) and measured Gal-3 levels following manufacturer’s recommendations as published previously by our team [25, 26]. In short, a pair of anti-Gal-3 antibodies were used in a sandwiched format to capture Gal-3 in the serum samples. The serum samples were diluted fourfold with 1% BSA in PBS. Colorimetric detection was achieved by using tetramethylbenzidine liquid substrate system.

### Development of NT-proBNP1–76aa and NT-proBNP13–71aa AlphaLISA assays

AlphaLISA immunoassays are bead-based homogeneous assays designed to study biomolecular interactions in a microplate format [15, 27]. We have previously validated the NT-proBNP_1-76aa_ AlphaLISA assay against a commercially available NT-proBNP_1-76aa_ diagnostic assay (Roche Diagnostics, USA) [28]. We found an agreement of *r*^2^ = 0.78 and *p* < 0.001. For quantification of the full-length NT-proBNP _1-76aa_, we have biotinylated mAB 5B6_1-12_ and conjugated mAb 24E11_67-76_ to acceptor beads (Product-No: 6772003, Perkin Elmer®, Waltham, MA, USA). Similarly, for quantifying NT-proBNP_13–71aa_ we have biotinylated mAb 15C4_63-71_ and conjugated mAb 18H5_13-20_ onto acceptor beads. Conjugation of antibodies to acceptor beads and the biotinylation of antibodies were carried out as previously published by us [15].

For the quantification of NT-proBNP full -length fragment and the NT-proBNP 13-71aa, 12-point standard curves were generated by titrating NT-proBNP analyte (Product-No: 8NT2, Hytest Ltd, Finland) in 1 × high block immunoassay buffer. In brief, 1 μL of either the analyte or sample was loaded in triplicates in a 384 well ProxiPlates™ (Perkin Elmer®, Waltham, MA, USA) and incubated with 5 μL of reagent containing acceptor beads (50 µg/mL) and biotinylated antibodies (5 nM) for 1.5 h at RT. Streptavidin Donor beads volume of 4 µL (80 µg/mL, Cat No.676002, Perkin Elmer®, Waltham, MA, USA) was added to the reaction mixture and incubated for 30-min at room temperature in the dark. The plates were read on EnVison™ plate reader (Perkin Elmer®, Waltham, MA, USA). The in-house AlphaLISA assays were previously validated against a commercial platform [28].

### Statistical analysis

Statistical analyses were performed with JMP Pro (V15.2.1, SAS Institute, Cary, NC, USA) and GraphPad Prism 8 (GraphPad Software Inc., La Jolla, California, USA). Graphpad was used to generate standard curves for Gal-3 by plotting the raw absorbance values on the Y-axis with the corresponding analyte concentrations. JMP was used for all other analyses. The analyte concentrations were deduced from the standard curves using four parameter logistic regression equation. Paired t-tests and correlations on log-transformed biomarkers were used to compare baseline and 1 month results. The primary endpoint, time to cardiovascular death or hospitalisation, was analysed using Cox proportional hazards regressions. Median splits on the biomarkers were used to display survival using Kaplan–Meier plots.

To assess the effect of covariates on the relationship between the biomarkers and the primary endpoint, a Cox regression model was used which included the covariates: NYHA functional class, presence of comorbidities (hypertension, diabetes, ischaemic heart disease (IHD), chronic obstructive pulmonary disease (COPD) or stroke), past history of HF, use of beta blockers, loop diuretics, digoxin and implantable cardioverter defibrillator in addition to the biomarkers, Gal-3 and NT-proBNP concentrations (Figs. 2). A Lasso penalised regression was applied to determine which covariates and predictors were most important for the cox model.

## Results

### Participants

A total of 101 patients with a primary diagnosis of HF were recruited to this observational, prospective study (Supplementary Table 1). We collected 101 samples at baseline and 48 samples at 1 month after the initial sampling (loss due to global Covid-19 pandemic lock-downs). Of these, 29 patients had events and 72 were censored, with a median follow up of 2.74 years (max 4.5 years). The majority of the patients were classified as NYHA class 2 and 3 patients (*n* = 89), with only 8 patients classified as NYHA class 1 and none was classified as NYHA class 4.

### The Gal-3 ELISA assay performance

We quantified the recovery of Gal-3 analyte using the in-house developed assay by spiking in Gal-3 at a concentrations of 1250 pg/mL and 350 pg/mL with recovery efficiencies of 97.6% and 101.8%, respectively. This recovery is acceptable for clinical studies [29]. The inter-assay variation was determined by repeated measurements of the same sample across three assays and was found to be 8.6% and the intra-assay variation was 1.5%. Both the intra- and inter-assay coefficient of variations are acceptable in a clinical diagnostic setting [30].

### The plasma NT-proBNP AlphaLISA® immunoassays performance

We determined the NT-proBNP_1-76aa_ and NTproBNP_13-71aa_ assay recoveries by spiking two known concentrations (100 pg/mL and 3000 pg/mL) of NT-proBNP analyte into pooled plasma samples_._ The spiked-in recovery was calculated by dividing the measured analyte concentration by the spiked in concentration. For NT-proBNP_1-76aa_ assay, the recovery was 73.20% and 92.79%, respectively, at 100 pg/mL and at 3000 pg/mL of spiked-in NT-proBNP. Similarly, the recoveries at 100 pg/mL was 94.31% and at 3000 pg/mL was 110.04% for NTproBNP_13-71aa_ assay. The lower limit of detection for the two assays were 5.79 pg/mL and 31.21 pg/mL, respectively. Both intra-and inter-assay coefficients of variations (%CV) were below 10%.

### The plasma concentrations of NT-proBNP1-76aa and NT-proBNP13-71aa in patients with HF

The baseline plasma concentrations of NT-proBNP_1-76aa_ and NTproBNP_13-71aa_ in patients with HF ranged between 0.45 and 2306 pg/mL with a median of 285.4 pg/mL (25% and 75% percentile: 99.05, 660.9 pg/mL, respectively) and 0–23,616 pg/mL with a median of 423.4 pg/mL (25% and 75% percentile: 112.2, 1120 pg/mL), respectively. The baseline concentrations of NTproBNP_13-71aa_ was significantly higher than NT-proBNP_1-76aa_ (paired *t*-test, *p* = 0.0033). The correlation coefficient between NTproBNP_13-71aa_ and NTproBNP_13-71aa_ using baseline samples indicated a moderate association (*r* = 0.57) (*p* < 0.0001).

At 1-month post-discharge, plasma NT-proBNP_1-76aa_ concentrations ranged from 0.91–2306 pg/mL with a median of 285.4 pg/mL (25% and 75% percentile: 58.37–669.6 pg/mL) at baseline versus 0–1769 pg/mL with a median of 182.7 pg/mL (25% and 75% percentile: 63.34–463 pg/mL) at 1 month; similarly, plasma NT-proBNP_13-71aa_ concentrations ranged from 0 to 3119 pg/mL with a median of 325.4 pg/mL (25% and 75% percentile: 112.2–1091 pg/mL) at baseline versus 0–9490 pg/mL with a median of 563.2 pg/mL (25% and 75% percentile: 103.1–1468 pg/mL) at 1 month. The concentrations of NTproBNP_13-71aa_ was significantly higher (paired *t*-test, *p* < 0.0001) than the concentrations of NT-proBNP_1-76aa_ at the 1-month follow-up. The correlation coefficient between NTproBNP_13-71aa_ and NTproBNP_13-71aa_ at 1 month showed a strong association (*r* = 0.81) (*p* < 0.001).

The concentrations of NT-proBNP_1-76aa_ at 1 month was significantly reduced from baseline (paired *t*-test, *p* = 0.0356), but the concentration of NT-proBNP_13-71aa_ fragments did not have statistically significant changes (paired *t*-test, *p* = 0.94). There was a strong correlation between baseline and 1-month NT-proBNP_1-76aa_ concentrations (*r* = 0.77, *p* < 0.0001) and for NTproBNP_13-71aa_ (*r* = 0.69, *p* < 0.0001).

### Gal-3 and NT-proBNP concentrations in patients with HF

**Fig. 1:**
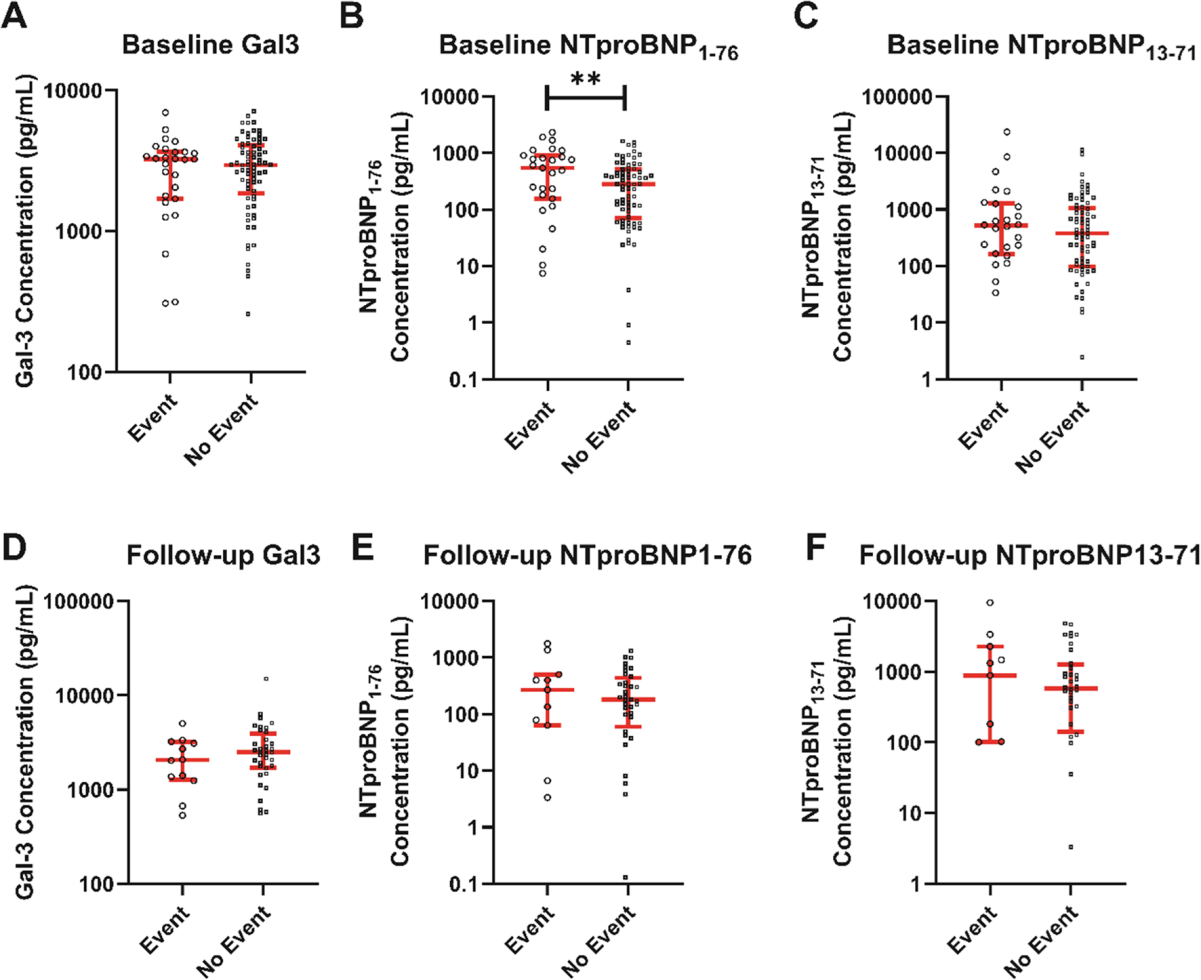
Median and Interquartile ranges with individual values of **A** baseline Gal-3 concentrations, **B** baseline NT-proBNP_1-76aa_ concentration, **C** baseline NT-proBNP_13-71aa_ concentration, **D** the changes of Gal-3 concentrations between 1 month post discharge and baseline, **E** the changes of NT-proBNP_1-76aa_ concentration between 1 month post discharge and baseline, **F** the changes of NT-proBNP_13-71aa_ concentration between 1 month post discharge and baseline in patients with and without primary endpoints

The primary endpoint, cardiovascular death or re-hospitalisations, occurred in 29 out of the 101 patients with HF within the follow up period (up to 4.5 years). When comparing patients with HF based on the median levels of baseline Gal-3, NT-proBNP_1-76_ and NT-proBNP_13-71_, we found that patients with events have significantly higher NT-proBNP_1-76aa_ concentrations when compared to patients without events (*p* < 0.01, Fig. 1B). While patients with higher Gal-3 and NT-proBNP_13-71,_ this was not significant (Fig. 1A, C). The delta concentration changes (at baseline and at 1 month) of Gal-3, NT-proBNP_1-76aa_ and NT-proBNP_13-71aa_, we found no statistical differences between patients with or without events (Fig. 1D, E, F). Time to cardiovascular death or re-hospitalisation is significantly shorter with increasing baseline NT-proBNP_1-76aa_ concentrations (Cox ph, Range HR = 12.1 95% CI (2.1, 58.8), *p* = 0.0058). Similarly, baseline NT-proBNP_13-71aa_ concentrations were also associated with shorter time to the primary endpoint (events), although this association was not significant (Cox PH, Range HR = 5.45, 95% CI 0.34, 32.32, *p* = 0.19). Similarly, baseline Gal-3 concentrations was also not associated with the primary endpoint (Cox PH, Range HR = 1.10, 95% CI 0.20, 5.59, *p* = 0.91).

**Fig. 2:**
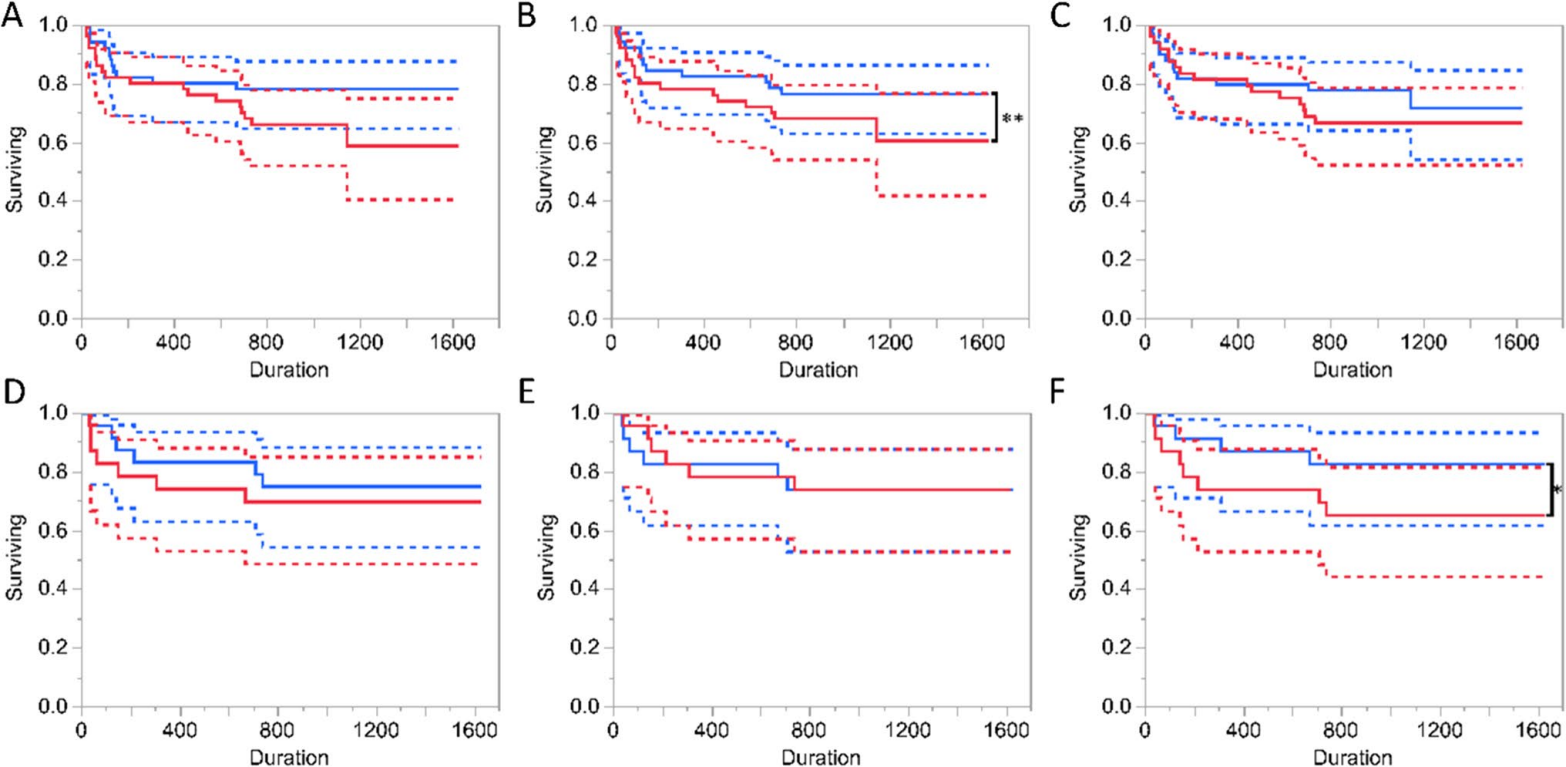
The Kaplan–Meier plot and the 95% confidence intervals (solid lines and dotted lines, respectively) for survival based on the median spilt of **A** Baseline Gal-3 concentrations, **B** Baseline NT-proBNP_1-76aa_ concentrations, **C** Baseline NT-proBNP_13-71aa_ concentrations, **D** the changes of Gal-3 concentrations between 1 month post discharge and baseline, **E** the changes of NT-proBNP_1-76aa_ concentrations between 1 month post discharge and baseline, **F** the changes of NT-proBNP_13-71aa_ concentrations between 1 month post discharge and baseline in patients. Patients with high biomarker levels are represented in red lines and patients with low biomarkers levels are represented with blue lines. Significant differences in survival were observed at baseline NT-proBNP_1-76aa_ concentrations and the changes of NT-proBNP_13-71aa_ concentrations between 1 month post discharge and baseline in patients with HF

We have also considered whether changes from baseline to 1-month follow-up would predict the time to the primary outcome using Gal-3 and NT-proBNP two fragments. However, at 1 month only 48 samples were available, comprising 13 patients with primary endpoint events and 35 patients with censored observations. Still, increase in NT-proBNP_13-71aa_ concentrations was associated with reduced time to the primary endpoint (HR = 64.2, 95% CI 2.47, 1671.5, *p* = 0.0123). The time to primary endpoint was not significantly associated with changes in Gal-3 or NT-proBNP_1-76aa_ (*p* = 0.18 and *p* = 0.24, respectively). To visualise this relationships, we present Kaplan–Meier plots of time to the primary endpoint versus median splits of the biomarkers (Fig. 2).

### The prognostic utility of Gal-3 and NT-proBNP concentrations based on Cox-regression analysis

The Cox proportional-hazard regression with double lasso model selection analysis was performed to evaluate the association between Gal-3, NT-proBNP fragments and time to the primary endpoint, controlling for covariates. The algorithm eliminates variables that do not contribute to the fit to Cox survival model. Covariates included in the model were age, sex, NYHA class, medical history, use of beta blockers, loop diuretics, or Digoxin, presence of comorbidities, including atrial fibrillation, hypertension, diabetes mellitus, IHD, COPD, and Stroke. The final model retained only Gal-3, NT-proBNP_1-76aa_ and NT-proBNP_13-71aa_ (Table 1).

**Table 1:** Parameter estimates for Gal-3, NT-proBNP1-76 and NT-proBNP13-71 at baseline in the cox survival analysis

Parameters	Estimate	Std. error	Wald Chi-square	Prob > Chi-square	Lower 95%	Upper 95%
Baseline Gal-3	8.0413e−5	7.6723e−5	1.0985085	0.2946	−0.00007	0.0002308
Baseline NT-proBNP_1-76aa_	0.0010137	0.0003012	11.330821	0.0008*	0.0004235	0.001604
Baseline NT-proBNP_13-71aa_	2.0023e−5	4.9437e−5	0.1640469	0.6855	−7.687e−5	0.0001169

## Discussion

To the best of our knowledge, this is one of the first studies to investigate the relationship between repeated measurements of Gal-3 and two fragment of NT-proBNP (NT-proBNP_1-76_ and NT-proBNP_13-71_) to predict cardiovascular deaths or rehospitalizations in HF patients. Our results demonstrated that HF patients with relatively high baseline NT-proBNP_1-76aa_ concentrations were 11 times more likely to reach the primary endpoint (cardiovascular deaths or rehospitalizations) even after adjusting for other covariates. Similarly, HF patients with relatively high baseline Gal-3 and NT-proBNP_13-71aa_ concentrations showed a worse prognosis, though not significant. We have chosen to investigate the concentrations of NT-proBNP fragments and Gal-3 as the former is involved during cardiac chamber dilation/stretch, and Gal-3 is a protein directly inducing pathologic remodelling of the heart, leading to cardiac fibrosis.

Our results demonstrated that baseline NT-proBNP_1-76aa_ concentrations was able to predict cardiovascular death or hospitalisation during the follow-up period. This is consistent with previous findings [31, 32], which reported that the natriuretic peptides are powerful predictors of all-cause mortality in patients with both chronic and acute HF. The findings from this study, along with previously published work from our group [15, 33] and others [34, 35] have demonstrated that there is molecular heterogeneity of NT-proBNP in plasma samples from patients with HF [36]. NT-proBNP antibodies used in some assays were often produced by raising against synthetic fragments of NT-proBNP with no post-translational modification, therefore, limiting the assay’s inability to detect endogenous forms. In addition, NT-proBNP could also be truncated at the glycosylation sites.

To improve current analytical challenges with full-length NT-proBNP assay, we investigated two different epitopes of the NT-proBNP molecule to stratify patients with HF. We have demonstrated that the diagnostic performance of the NT-proBNP_13-71aa_ immunoassay showed improvements over the commercial NT-proBNP_1-76aa_ assay based on previously published literature (pooled sensitivity and specificity 80% and 61% respectively [12]). This is because we have chosen antibodies directed away from the N and C termini (truncated) as well as avoiding the glycosylated regions within the NT-ProBNP molecule [15, 16]. We have found that we can detect NT-proBNP_13-71aa_ in a much higher abundance in comparison to NT-proBNP_1-76aa_, which indicated the high frequency of endogenous cleavage of threonine at 71 a.a. The utilization of NT-proBNP_13-71aa_ potentially can improve the prognostic utility of NT-proBNP as it is more sensitive than the standard assay and may therefore more reliably reflect disease severity.

Our results demonstrated that the median Gal-3 concentrations in patients with HF who reached the primary endpoints was 3237 pg/mL while the patients without a primary endpoint had a medium Gal-3 concentration of 2946 pg/mL (not significant). We were unable to find a significant correlation between rehospitalisation and Gal-3 concentrations, however the Cox Proportional Hazards analysis with step-wise double lasso validation suggested that Gal-3 concentrations still played an important role in developing a model to discriminate patients with and without adverse events. Similarly, the Coordinating study evaluating Outcomes of Advising and Counselling in HF (COACH) revealed that the higher levels of Gal-3 in blood were associated with a greater risk of death or re-hospitalisation due to HF [10]. In their study, blood Gal-3 levels demonstrated an added prognostic power over B-type natriuretic peptide (BNP). Furthermore, the Deventer-Alkmaar HF (DEAL-HF) study that followed patients with chronic HF for up to 6.5 years, demonstrated that blood Gal-3 levels were a significant predictor of mortality even after adjusting for age, sex and the known risk factors [11]. However, this study’s analysis was limited to mortality, and did not take into account re-hospitalisation due to HF. The TRIple Pill vs. Usual care Management for Patients with mild- to- moderate Hypertension (TRIUMPH) study evaluated 496 patients with HF over 325 days and found a weak association between single blood Gal-3 measurements and subsequent death or HF hospitalisation [37]. In contrast, when repeated measures were considered, the adjusted hazard ratio was 1.67 (95% confidence interval (CI) 1.24—2.23, *p* < 0.001) per one standard deviation increase in Gal-3, which remained significant after adjusting for NTproBNP [38].

There are number of limitations to this study. In this study, we have only included cardiovascular rehospitalisation and death as the primary endpoints. We chose not to use heart failure rehospitalisation as a primary endpoint due to lower rates of rehospitalisation compared to cardiovascular rehospitalisation in our study cohort. Physiologically, Gal-3 is relevant to cardiovascular heath in general rather than specific to HF and investigating whether Gal-3 can be used to predict the cardiovascular rehospitalisation is more relevant in the current clinical setting. We also did not use all-cause mortality as an endpoint to increase the specificity and clinical relevance of our findings to identify patients at high risk of cardiovascular events. We were also unable to collect sample from half of the recruited patient at the follow-up time point due to Covid-19 lock downs in Australia. Future larger studies could investigate whether Gal-3 is associated with non-cardiovascular events (e.g. infection-related adverse events). Due to the differences in routine clinical management and collection of clinical data across the three collection sites, the clinical observation and clinical tests were not collected uniformly throughout the study. This limited the number of cofounding factors that we could investigate in this study. In addition, future studies will need to determine whether repeated sampling of serum Gal-3 can improve its prognostic utility as well as to include both HFpEF and HFrEF patients.

## Conclusion

In summary, we have demonstrated that Gal-3 and NT-proBNP concentrations can be used as prognostic biomarkers to stratify patients with HF even after adjusting for other confounding variables. While these findings require confirmation in larger multi-centred clinical studies, the long-term monitoring of the combination of these biomarkers could potentially improve the management of HF in a community setting.

### Supplementary Information

Below is the link to the electronic supplementary material.

Supplementary file1 (DOCX 104 KB)

## Data Availability

All the data produced and used in this manuscript are included in Supplementary Table. All authors take responsibility for all aspects of the reliability and freedom from bias of the data presented and their discussed interpretation.

## References

[1] James SL (2018). Global, regional, and national incidence, prevalence, and years lived with disability for 354 diseases and injuries for 195 countries and territories, 1990–2017: a systematic analysis for the Global Burden of Disease Study 2017. Lancet.

[2] Krumholz HM, Merrill AR, Schone EM, Schreiner GC, Chen J, Bradley EH, Wang Y, Wang Y, Lin Z, Straube BM, Rapp MT, Normand SL, Drye EE (2009). Patterns of hospital performance in acute myocardial infarction and heart failure 30-day mortality and readmission. Circ Cardiovasc Qual Outcomes.

[3] Tromp J, Bamadhaj S, Cleland JGF, Angermann CE, Dahlstrom U, Ouwerkerk W, Tay WT, Dickstein K, Ertl G, Hassanein M, Perrone SV, Ghadanfar M, Schweizer A, Obergfell A, Lam CSP, Filippatos G, Collins SP (2020). Post-discharge prognosis of patients admitted to hospital for heart failure by world region, and national level of income and income disparity (REPORT-HF): a cohort study. Lancet Glob Health.

[4] Wettersten N, Maisel A (2015). Role of cardiac troponin levels in acute heart failure. Card Fail Rev.

[5] Wang Z, Pan X, Xu H, Wu Y, Jia X, Fang Y, Lu Y, Xu Y, Zhang J, Su Y (2022). Serum soluble ST2 is a valuable prognostic biomarker in patients with acute heart failure. Front Cardiovasc Med.

[6] Lakhani I, Wong MV, Hung JKF, Gong M, Waleed KB, Xia Y, Lee S, Roever L, Liu T, Tse G, Leung KSK, Li KHC (2021). Diagnostic and prognostic value of serum C-reactive protein in heart failure with preserved ejection fraction: a systematic review and meta-analysis. Heart Fail Rev.

[7] Voors AA, Kremer D, Geven C, Ter Maaten JM, Struck J, Bergmann A, Pickkers P, Metra M, Mebazaa A, Düngen HD, Butler J (2019). Adrenomedullin in heart failure: pathophysiology and therapeutic application. Eur J Heart Fail.

[8] Sharma UC, Pokharel S, van Brakel TJ, van Berlo JH, Cleutjens JP, Schroen B, Andre S, Crijns HJ, Gabius HJ, Maessen J, Pinto YM (2004). Galectin-3 marks activated macrophages in failure-prone hypertrophied hearts and contributes to cardiac dysfunction. Circulation.

[9] Heymans S, Gonzalez A, Pizard A, Papageorgiou AP, Lopez-Andres N, Jaisser F, Thum T, Zannad F, Diez J (2015). Searching for new mechanisms of myocardial fibrosis with diagnostic and/or therapeutic potential. Eur J Heart Fail.

[10] de Boer RA, Lok DJ, Jaarsma T, van der Meer P, Voors AA, Hillege HL, van Veldhuisen DJ (2011). Predictive value of plasma galectin-3 levels in heart failure with reduced and preserved ejection fraction. Ann Med.

[11] Lok DJ, Van Der Meer P, de la Porte PW, Lipsic E, Van Wijngaarden J, Hillege HL, van Veldhuisen DJ (2010). Prognostic value of galectin-3, a novel marker of fibrosis, in patients with chronic heart failure: data from the DEAL-HF study. Clin Res Cardiol.

[12] Salah K, Stienen S, Pinto YM, Eurlings LW, Metra M, Bayes-Genis A, Verdiani V, Tijssen JGP, Kok WE (2019). Prognosis and NT-proBNP in heart failure patients with preserved versus reduced ejection fraction. Heart.

[13] Spinar J, Spinarova L, Malek F, Ludka O, Krejci J, Ostadal P, Vondrakova D, Labr K, Spinarova M, Pavkova Goldbergova M, Benesova K, Jarkovsky J, Parenica J (2019). Prognostic value of NT-proBNP added to clinical parameters to predict two-year prognosis of chronic heart failure patients with mid-range and reduced ejection fraction—a report from FAR NHL prospective registry. PLoS ONE.

[14] Ala-Kopsala M, Magga J, Peuhkurinen K, Leipälä J, Ruskoaho H, Leppäluoto J, Vuolteenaho O (2004). Molecular heterogeneity has a major impact on the measurement of circulating N-terminal fragments of A- and B-type natriuretic peptides. Clin Chem.

[15] Foo JY, Wan Y, Schulz BL, Kostner K, Atherton J, Cooper-White J, Dimeski G, Punyadeera C (2013). Circulating fragments of N-terminal pro-B-type natriuretic peptides in plasma of heart failure patients. Clin Chem.

[16] Halfinger B, Hammerer-Lercher A, Amplatz B, Sarg B, Kremser L, Lindner HH (2017). Unraveling the molecular complexity of O-glycosylated endogenous (N-terminal) pro–B-type natriuretic peptide forms in blood plasma of patients with severe heart failure. Clin Chem.

[17] Heidenreich PA, Albert NM, Allen LA, Bluemke DA, Butler J, Fonarow GC, Ikonomidis JS, Khavjou O, Konstam MA, Maddox TM, Nichol G, Pham M, Pina IL, Trogdon JG, American Heart Association Advocacy Coordinating C, Council on Arteriosclerosis T, Vascular B, Council on Cardiovascular R, Intervention Council on Clinical C, Council on E Prevention Stroke C (2013). Forecasting the impact of heart failure in the United States: a policy statement from the American Heart Association. Circ Heart Fail.

[18] van Walraven C, Jennings A, Forster AJ (2012). A meta-analysis of hospital 30-day avoidable readmission rates. J Eval Clin Pract.

[19] Virani SS, Alonso A, Benjamin EJ, Bittencourt MS, Callaway CW, Carson AP, Chamberlain AM, Chang AR, Cheng S, Delling FN, Djousse L, Elkind MSV, Ferguson JF, Fornage M, Khan SS, Kissela BM, Knutson KL, Kwan TW, Lackland DT, Lewis TT, Lichtman JH, Longenecker CT, Loop MS, Lutsey PL, Martin SS, Matsushita K, Moran AE, Mussolino ME, Perak AM, Rosamond WD, Roth GA, Sampson UKA, Satou GM, Schroeder EB, Shah SH, Shay CM, Spartano NL, Stokes A, Tirschwell DL, VanWagner LB, Tsao CW, American Heart Association Council on E, Prevention Statistics C, Stroke Statistics S (2020). Heart disease and stroke statistics-2020 update: a report from the American Heart Association. Circulation.

[20] Writing Committee M, Yancy CW, Jessup M, Bozkurt B, Butler J, Casey DE, Drazner MH, Fonarow GC, Geraci SA, Horwich T, Januzzi JL, Johnson MR, Kasper EK, Levy WC, Masoudi FA, McBride PE, McMurray JJ, Mitchell JE, Peterson PN, Riegel B, Sam F, Stevenson LW, Tang WH, Tsai EJ, Wilkoff BL, A College of Cardiology Foundation, American Heart Association Task Force on Practice G (2013). ACCF/AHA guideline for the management of heart failure: a report of the American College of Cardiology Foundation/American Heart Association Task Force on practice guidelines. Circulation.

[21] World Medical Association (2013) World Medical Association Declaration of Helsinki: ethical principles for medical research involving human subjects. Jama 310(20):2191–2194. 10.1001/jama.2013.281053. PMID: 24141714 10.1001/jama.2013.281053 24141714

[22] Australian Research Council and Universities Australia (2018). Australian code for responsible conduct of research, R41.

[23] Atherton JJ, Sindone A, De Pasquale CG, Driscoll A, MacDonald PS, Hopper I, Kistler PM, Briffa T, Wong J, Abhayaratna W, Thomas L, Audehm R, Newton P, O'Loughlin J, Branagan M, Connell C (2018). National Heart Foundation of Australia and Cardiac Society of Australia and New Zealand: Guidelines for the Prevention, Detection, and Management of Heart Failure in Australia 2018. Heart Lung Circ.

[24] Dolgin M, Committee NYHAC (1994). Nomenclature and criteria for diagnosis of diseases of the heart and great vessels.

[25] R&D Systems Human Galectin-3 DuoSet ELISA (Cat#DY1154) manual, obtianed from https://www.rndsystems.com/products/human-galectin-3-duoset-elisa_dy1154

[26] Zhang X, Dimeski G, Punyadeera C (2014). Validation of an immunoassay to measure plasminogen-activator inhibitor-1 concentrations in human saliva. Biochem Med (Zagreb).

[27] Soleh MT, Foo JY, Bailey UM, Tan NY, Wan Y, Cooper-White J, Schulz BL, Punyadeera C (2014). A rapid and cost-effective method of producing recombinant proBNP and NT-proBNP variants in *Escherichia coli* for immunoassay of heart failure. Biotech Lett.

[28] Foo JY, Wan Y, Kostner K, Arivalagan A, Atherton J, Cooper-White J, Dimeski G, Punyadeera C (2012). NT-ProBNP levels in saliva and its clinical relevance to heart failure. PLoS ONE.

[29] Rifai N, Gillette MA, Carr SA (2006). Protein biomarker discovery and validation: the long and uncertain path to clinical utility. Nat Biotechnol.

[30] Sittampalam GS, Coussens NP, Brimacombe K, Grossman A, Arkin M, Auld D, Austin C, Baell J, Bejcek B, Caaveiro JMM, Chung TDY, Dahlin JL, Devanaryan V, Foley TL, Glicksman M, Hall MD, Haas JV, Inglese J, Iversen PW, Kahl SD, Kales SC, Lal-Nag M, Li Z, McGee J, McManus O, Riss T, Trask OJ, Jr., Weidner JR, Wildey MJ, Xia M, Xu X (2004) Assay guidance manual. Eli Lilly & Company and the National Center for Advancing Translational Sciences, Bethesda 22553861

[31] Hartmann F, Packer M, Coats AJ, Fowler MB, Krum H, Mohacsi P, Rouleau JL, Tendera M, Castaigne A, Trawinski J, Amann-Zalan I, Hoersch S, Katus HA (2004). NT-proBNP in severe chronic heart failure: rationale, design and preliminary results of the COPERNICUS NT-proBNP substudy. Eur J Heart Fail.

[32] Yu CM, Sanderson JE (1999). Plasma brain natriuretic peptide—an independent predictor of cardiovascular mortality in acute heart failure. Eur J Heart Fail.

[33] Wan Y, Xhang X, Atherton JJ, Kostner K, Dimeski G, Punyadeera C (2014). A multimarker approach to diagnose and stratify heart failure. Int J Cardiol.

[34] Apple FS (2005). Standardization of cardiac markers. Scand J Clin Lab Invest Suppl.

[35] Apple FS, Panteghini M, Ravkilde J, Mair J, Wu AH, Tate J, Pagani F, Christenson RH, Jaffe AS, Committee on Standardization of Markers of Cardiac Damage of the I (2005). Quality specifications for B-type natriuretic peptide assays. Clin Chem.

[36] Seferian KR, Tamm NN, Semenov AG, Tolstaya AA, Koshkina EV, Krasnoselsky MI, Postnikov AB, Serebryanaya DV, Apple FS, Murakami MM, Katrukha AG (2008). Immunodetection of glycosylated NT-proBNP circulating in human blood. Clin Chem.

[37] van Vark LC, Lesman-Leegte I, Baart SJ, Postmus D, Pinto YM, de Boer RA, Asselbergs FW, Wajon E, Orsel JG, Boersma E, Hillege HL, Akkerhuis KM, Investigators T (2017). Prognostic value of serial galectin-3 measurements in patients with acute heart failure. J Am Heart Assoc.

[38] Motiwala SR, Szymonifka J, Belcher A, Weiner RB, Baggish AL, Sluss P, Gaggin HK, Bhardwaj A, Januzzi JL (2013). Serial measurement of galectin-3 in patients with chronic heart failure: results from the ProBNP Outpatient Tailored Chronic Heart Failure Therapy (PROTECT) study. Eur J Heart Fail.

