# Supplementary material for: Prognostic utility of serum NT-proBNP (fragments 1-76aa and 13-71aa) and galectin-3 in predicting death and re-hospitalisation due to cardiovascular events in patients with heart failure
Source: Heart Vessels. 2023 Aug 3;39(1):86–94. doi: 10.1007/s00380-023-02296-z (PMC10764512; doi:10.1007/s00380-023-02296-z)
Supplement: Supplementary file 1 — Supplementary file1 (DOCX 104 KB) [file 380_2023_2296_MOESM1_ESM.docx]

|  | Demographic History | | | | | | Etiology | | | Other Mediacl coditions | | | | | | | NYHA Classification | Vital Signs | | | ECG and Echocardiogram Findings | | | Biochemistry | | | | | | | | | Treatments | | | | | | Events (cardiovascular death or rehospitalisation due to HF) | | |
| --- | --- | --- | --- | --- | --- | --- | --- | --- | --- | --- | --- | --- | --- | --- | --- | --- | --- | --- | --- | --- | --- | --- | --- | --- | --- | --- | --- | --- | --- | --- | --- | --- | --- | --- | --- | --- | --- | --- | --- | --- | --- |
| Clinical Data | Age | Sex | Current smoker (Yes/No) | Exsmoker (Yes) | Never Smoked | Alcohol intake (Yes/No/Ex-drinker) | Hypertention | Ischemic heart disease (IHD) | Valuar heart disease (VHD) | Diabetes mellitus | Dyslipidemia | Chronic Obstructive Pulmonary Disease (COPD) | Stroke | Past medical history of HF | Pleural effusion | Peripheral oedema | NYHA functional Class (1,2,3,4) | Heart rate | Systolic blood pressure (mmHg) | Diastolic blood pressure (mmHg) | QRS ECG | Atrial fibrilation | Left ventricular ejection fraction (LVEF) % | Hb (g/dL) | Leucocyte count (10E9/L) | Neutrophils count (10E9/L) | Lymphocytes count (10E9/L) | Sodium (mM/L) | Potasium (mM/L) | Creatinine (µM/L) | Urea (mM/L) | eGFR (mL/ min) | ACEI or ARB | Beta-blocker | Spironolatone/Eplerenone | Loop Diuretic | Digoxin | Intra cardiac device (ICD) | Date of collection | Event within 5 years |  |
| Mater - HF-1 | 89 | Male |  |  | Yes | No | Yes | Yes | No | No | Yes | No | No | No | No | Yes | 2 | 90 | 120 | 60 |  | Yes | 50-55% | 127 | 5.1 | 3.87 | 0.61 | 139 | 4.2 | 77 | 11.9 | 77 | Yes | Yes | No | Yes | Yes | No | 29.06.2016 | CV hospitalisation (03.11.2016) |  |
| Mater -HF-10 | 50 | Male | Yes |  |  | Yes | Yes | No | No | Yes | Yes | No | No | Yes | No | No | 2,3 | 60 | 108 | 68 |  | No | 20-25% | NA | NA | NA | NA | NA | NA | NA | NA | NA | Yes | No | Yes | Yes | No | No | 11.07.2017 | No |  |
| Mater -HF-12 | 53 | Female |  | Yes |  | No | Yes | No | No | Yes | Yes | No | Yes | Yes | No | No | 2,3 | 60 | 120 | 70 |  | No | 25% | 126 | 8.1 | 4.43 | 2.7 | 136 | 4.5 | 73 | 7.5 | 80 | No | Yes | Yes | Yes | No | No | 08.08.2017 | No |  |
| Mater -HF-13 | 48 | Female | Yes |  |  | No | No | No | No | No | No | Yes | No | No | No | No | 3 | 100 | 100 | 70 |  | No | 15-20% | 152 | 12.2 | 9.59 | 1.3 | 138 | 3.5 | 57 | 4.1 | >90 | Yes | Yes | No | Yes | Yes | No | 17.08.2017 | No |  |
| Mater -HF-14 | 60 | Female | No |  |  | No | No | No | No | No | No | No | No | No | No | No | 2 | 90 | 100 | 60 |  | No | 20% | 134 | 9.1 | 4.49 | 2.99 | 143 | 4.8 | 91 | 8.5 | 59 | No | Yes | Yes | No | No | No | 28.8.2017 | No |  |
| Mater -HF-15 | 70 | Male |  | Yes |  | No | Yes | Yes | No | No | Yes | No | No | Yes | No | No | 2 | NA | 122 | 64 |  | No | 35-40% | NA | NA | NA | NA | NA | NA | NA | NA | NA | Yes | Yes | Yes | No | No | No | 28.09.2017 | No |  |
| Mater -HF-16 | 71 | Male | No |  |  | No | Yes | No | No | Yes | Yes | Yes | No | Yes | Yes | No | 3 | 60 | NA | NA |  | No | 35-40% | NA | NA | NA | NA | NA | 134 | 113 | 11.1 | 56 | Yes | Yes | No | No | No | No | 12.10.2017 | CV hospitalisation (27.12.2018) |  |
| Mater -HF-17 | 79 | Male |  | Yes |  | No | Yes | Yes | No | No | Yes | No | No | Yes | Yes | No | 2,3 | 80 | 120 | 96 |  | Yes | 25-30% | 154 | 9.2 | 5.32 | 2.43 | 142 | 4 | 106 | 11.2 | 57 | Yes | Yes | No | Yes | No | No | 27.10.2017 | No |  |
| Mater -HF-18 | 39 | Male | No |  |  | No | No | No | No | Yes | No | No | No | No | Yes | No |  | 90 | 90 | 60 |  | No | 10% | 170 | 7.3 | 4.31 | 1.76 | 140 | 4.4 | 96 | 9.4 | 86 | Yes | Yes | Yes | Yes | No | No | 10.11.2017 | No |  |
| Mater -HF-2 | 84 | Male | No |  |  | Ex | Yes | Yes | No | No | Yes | No | No | No | No | No | 2, 3 | 82 | 120 | 65 |  | No | 40-45% | 100 | 9.4 | 6.43 | 1.42 | 140 | 3.5 | 79 | 15.4 | 62 | No | No | No | Yes | No | No | 10.10.2016 | CV hospitalisation (26.08.2018) |  |
| Mater -HF-3 | 72 | Female | No |  |  | No | Yes | No | Yes | Yes | Yes | Yes | No | No | NA | NA | 3 | 62 | 130 | 64 |  | Yes | 60% | 91 | 7.3 | 3.93 | 2.28 | 140 | 3.9 | 111 | 13 | 43 | No | Yes | No | Yes | No | Yes | 10.10.2016 | No |  |
| Mater -HF-4 | 54 | Male | No |  |  | No | Yes | Yes | No | No | No | No | Yes | No | No | No | 2 | 60 | 130 | 70 |  | No | 55-60% | 125 | 11.5 | 7.56 | 2.08 | 145 | 309 | 148 | 13.1 | 46 | Yes | Yes | No | No | No | No | 02.05.2017 | No |  |
| Mater -HF-5 | 80 | Female | No |  |  | No | No | No | No | No | Yes | No | No | No | NA | NA | 2,3 | 50 | 120 | 60 |  | No | 24% | 133 | 9.3 | 5.38 | 2.58 | 139 | 4.3 | 109 | 6.5 | 41 | No | Yes | No | Yes | No | No | 02.05.2017 | No |  |
| Mater -HF-6 | 43 | Male | Yes |  |  | Yes | No | No | No | No | No | No | No | No | NA | NA | 2,3 | 106 | 92 | 70 |  | No | 10-15% | 141 | 8 | 4.79 | 2.06 | 133 | 3.6 | 69 | 6.5 | >90 | Yes | Yes | Yes | Yes | No | No | 19.05.2017 | CV hospitalisation (27.06.2017) |  |
| RBWG-HF-24 | 82 | Male | No |  |  | No | No | No | No | No | No | No | No | Yes | No | No | 1 | 66 | 120 | 60 |  | No | 15% | NA | NA | NA | NA | NA | NA | NA | NA | NA | Yes | Yes | Yes | No | No | Yes | 10.04.2017 | No |  |
| RBWH-HF -10 | 85 | Female | Yes |  |  | NA | Yes | Yes | No | No | Yes | Yes | No | Yes | Yes | No | 3 | 80 | 120 | 80 |  | No | 45% | NA | NA | NA | NA | NA | NA | NA | NA | NA | Yes | Yes | No | Yes | Yes | Yes | 14.04.2016 | No |  |
| RBWH-HF -10-2 | 44 | Male |  |  |  | NA | NA | NA | NA | NA | NA | NA | NA | NA | NA | NA | 2 | NA | NA | NA |  | No | 34% | NA | NA | NA | NA | NA | NA | NA | NA | NA | NA | NA | NA | NA | NA | NA | 02.07.2016 | No |  |
| RBWH-HF -11 | 51 | Male | No |  |  | No | Yes | Yes | No | Yes | Yes | No | No | Yes | No | No | 2 | NA | NA | NA |  | Yes | 30% | NA | NA | NA | NA | NA | NA | NA | NA | NA | No | Yes | Yes | Yes | Yes | Yes | 18.07.2016 | No |  |
| RBWH-HF -13 | 49 | Male |  |  | Yes | No | No | No | No | No | No | No | No | No | No | No | 2 | NA | NA | NA |  | No | 30% | NA | NA | NA | NA | NA | NA | NA | NA | NA | Yes | Yes | Yes | No | No | No | 01.08.2016 | No |  |
| RBWH-HF -14 | 75 | Female |  | Yes |  | No | Yes | Yes | Yes | No | Yes | Yes | No | Yes | No | No | 3 | 70 | 170 | 70 |  | No | 40-45% | NA | NA | NA | NA | NA | NA | NA | NA | NA | Yes | No | No | No | No | No | 04.08.2016 | No |  |
| RBWH-HF -15 | 76 | Male |  |  | Yes | No | Yes | Yes | No | No | No | No | No | Yes | NA | NA | 2 | 59 | 110 | 75 |  | No | 32% | NA | NA | NA | NA | NA | NA | NA | NA | NA | No | Yes | Yes | Yes | No | No | 31.10.2017 | No |  |
| RBWH-HF -18 | 80 | Female | No |  |  | No | Yes | Yes | Yes | Yes | Yes | No | No | Yes | Yes | Yes | 2 | 65 | 110 | 60 |  | Yes | 20-25% | 129 | 7.4 | 4.76 | 1.57 | 136 | 4.4 | 64 | 7.4 | 79 | No | Yes | Yes | No | No | Yes | 13.02.2017 | No |  |
| RBWH-HF -1 | 69 | Female | No |  |  | No | Yes | No | No | No | No | No | No | Yes | No | No | 2 | 65 | 160 | 90 |  | Yes | 35-40% | NA | NA | NA | NA | NA | NA | NA | NA | NA | Yes | Yes | No | No | No | No | 04.02.2016 | No |  |
| RBWH-HF -2 | 53 | Female | No |  |  | No | No | Yes | No | NA | NA | NA | NA | NA | NA | NA | 2 | NA | NA | NA |  | No | 30-35% | NA | NA | NA | NA | NA | NA | NA | NA | NA | NA | NA | NA | NA | NA | NA | 04.02.2016 | No |  |
| RBWH-HF -3 | 71 | Male | No |  |  | No | No | Yes | No | No | No | No | No | Yes | No | No | 2 | NA | NA | NA |  | Yes | 45-50% | NA | NA | NA | NA | NA | NA | NA | NA | NA | Yes | Yes | Yes | No | Yes | Yes | 04.02.2016 | No |  |
| RBWH-HF -4 | 64 | Male |  | Yes |  | No | Yes | No | No | Yes | No | Yes | No | No | No | No | 2 | NA | NA | NA | LBBB/ AV block | No | 15-20% | NA | NA | NA | NA | NA | NA | NA | NA | NA | No | Yes | Yes | Yes | No | Yes | 04.02.2016 | CV hospitalisation (07.12.2016) |  |
| RBWH-HF -5 | 86 | Male | No |  |  | No | Yes | Yes | No | No | Yes | No | No | Yes | No | No | 2 | NA | NA | NA |  | No | 37% | NA | NA | NA | NA | NA | NA | NA | NA | NA | No | Yes | Yes | No | No | Yes | 18.02.2016 | No |  |
| RBWH-HF -6 | 67 | Male |  | Yes |  | No | No | Yes | No | Yes | Yes | Yes | No | Yes | No | No | 3 | NA | NA | NA |  | Yes | 35-40% | 139 | 7.6 | 5.11 | 1.68 | 137 | 4.3 | 112 | 9.2 | 59 | Yes | Yes | Yes | Yes | No | No | 03.03.2016 | CV hospitalisation(04.04.2016) |  |
| RBWH-HF -7 | 98 | Female | No |  |  | No | No | Yes | No | No | Yes | No | No | No | No | No | 3 | NA | NA | NA | NA | NA | 59% | 106 | 54.3 | 7.11 | 45.97 | 138 | 4.2 | 138 | 14.9 | 63 | No | Yes | Yes | No | No | No | 03.03.2016 | CV hospitalisation (01.08.2016) |  |
| RBWH-HF -8 | 90 | Female | No |  |  | No | Yes | Yes | No | Yes | Yes | No | Yes | Yes | No | No | 2 | 55 | 120 | 70 |  | Yes | 24% | NA | NA | NA | NA | NA | NA | NA | NA | NA | No | Yes | No | No | No | No | 03.03.2016 | No |  |
| RBWH-HF -9 | 54 | Male | No |  |  | EX | Yes | Yes | No | No | Yes | No | No | Yes | No | No | 2 | NA | NA | NA |  | No | 40-45% | NA | NA | NA | NA | NA | NA | NA | NA | NA | Yes | Yes | Yes | Yes | No | No | 24.03.2016 | No |  |
| RBWH-HF-19 | 76 | Male |  |  | Yes | Ex | Yes | Yes | No | No | Yes | No | No | Yes | No | No | 2,3 | 90 | 100 | 45 |  | Yes | 25-30% | 121 | 6.6 | 4.26 | 1.52 | 140 | 5 | 126 | 6.8 | 47 | No | Yes | Yes | Yes | No | Yes | 16.02.2017 | CV hospitalisation (22.05.2018) |  |
| RBWH-HF-21 | 78 | Female | No |  |  | No | Yes | No | No | No | No | No | No | Yes | No | No | 3 | 50 | 160 | 75 |  | No | 20-25% | NA | NA | NA | NA | NA | NA | NA | NA | NA | Yes | Yes | No | Yes | No | No | 23.02.2017 | No |  |
| RBWH-HF-22 | 97 | Female | No |  |  | No | Yes | Yes | No | No | Yes | No | No | Yes | No | No | 3 | 65 | 110 | 60 |  | No | 30-35% | NA | NA | NA | NA | NA | NA | NA | NA | NA | Yes | No | No | Yes | No | No | 23.02.2017 | CV hospitalisation (14.04.2020) |  |
| RBWH-HF-23 | 62 | Male |  | Yes |  | No | Yes | Yes | No | No | Yes | No | No | Yes | No | No | 1 | 65 | 110 | 100 |  | No | 30-35% | NA | NA | NA | NA | NA | NA | NA | NA | NA | No | Yes | Yes | Yes | No | Yes | 10.04.2017 | No |  |
| RBWH-HF-25 | 64 | Male | No |  |  | No | Yes | Yes | No | Yes | Yes | No | No | Yes | No | No | 2 | NA | Na | NA |  | No | 35-40% | 120 | 6.4 | 7.93 | 3.2 | 136 | 4.7 | 182 | 31.2 | 33 | Yes | Yes | No | Yes | No | No | 10.05.2017 | No |  |
| RBWH-HF-27 | 88 | Female | No |  |  | No | Yes | No | No | No | No | Yes | No | No | No | No | 2 | NA | NA | NA |  | No | 35% | 114 | 6.1 | 3.85 | 0.93 | 135 | 4.2 | 63 | 4.2 | 79 | Yes | Yes | No | Yes | No | No | 22.05.2017 | No |  |
| RBWH-HF-28 | 62 | Male | No |  |  | No | Yes | Yes | No | Yes | Yes | Yes | No | Yes | Yes | Yes | 2 | NA | NA | NA |  | Yes | 35% | 102 | 6.1 | 3.52 | 1.3 | 142 | 3.7 | 295 | 19.1 | 19 | Yes | Yes | Yes | No | Yes | No | 06.06.2017 | CV hospitalisation(26.11.2017) | CV death (04.01.2018) |
| RBWH-HF-29 | 51 | Male | No |  |  | No | No | No | No | No | No | No | No | No | No | No | 2 | NA | NA | NA |  | Yes | 24% | 167 | 7.1 | 4.68 | 0.95 | 139 | 4.6 | 107 | 10.1 | 70 | Yes | Yes | Yes | Yes | No | No | 19.06.2017 | Rehospitalisation due to HF (27.07.2017) |  |
| RBWH-HF-30 | 72 | Female |  |  | No | No | No | Yes | No | Yes | Yes | No | No | Yes | No | No | 3 | NA | NA | NA |  | No | 25% | 101 | 3.8 | 2.12 | 1.6 | 143 | 4 | 148 | 13 | 30 | No | Yes | No | Yes | No | No | 22.06.2017 | No |  |
| RBWH-HF-31 | 64 | Male | No |  |  | No | No | No | No | No | No | No | No | No | No | No | 1 | NA | NA | NA |  | No | 18% | 156 | 8.6 | 4.37 | 3.12 | 140 | 4.5 | 88 | 8.1 | 63 | Yes | Yes | Yes | Yes | No | Yes | 17.07.2017 | No |  |
| RBWH-HF-32 | 77 | Male | No |  |  | No | Yes | No | No | Yes | Yes | No | No | No | No | No | 1 | NA | NA | NA |  |  | 40% | 120 | 5.5 | 3.82 | 0.72 | 135 | 4.4 | 126 | 8.7 | 47 | No | Yes | Yes | No | No | No | 27.07.2017 | No |  |
| RBWH-HF-33 | 51 | Male | No |  |  | No | No | No | No | No | No | No | No | No | No | No | 1 | NA | NA | NA |  | Yes | 35-40% | 154 | 6.7 | 4.08 | 1.76 | 138 | 3.8 | 91 | 6 | 84 | Yes | Yes | No | No | No | No | 27.07.2017 | No |  |
| RBWH-HF-34 | 69 | Male | No |  |  | No | Yes | Yes | No | Yes | Yes | No | No | No | No | No | 2 | NA | NA | NA |  | No | 35% | 104 | 5.2 | 3.56 | 0.98 | 129 | 5.5 | 134 | 20.6 | 38 | No | Yes | Yes | Yes | Yes |  | 10.08.2017 | No |  |
| RBWH-HF-35 | 75 | Male | No |  |  | No | No | Yes | No | No | No | No | No | No | No | No | 2 | NA | NA | NA |  | Yes | 35% | 169 | 8.5 | 5.45 | 1.63 | 138 | 3.4 | 104 | 10 | 68 | No | Yes | No | Yes | No |  | 21.09.2017 | No |  |
| RBWH-HF-37 | 56 | Male | Yes |  |  | Yes | No | No | No | No | No | No | No | No | No | No | 1 | 60 | 100 | 65 |  | No | 20% | 129 | 7.5 | 4.41 | 2.23 | 136 | 4.4 | 87 | 5 | 62 | No | Yes | Yes | Yes | No | Yes | 05.10.2017 | CV hospitalisation (07.12.2017) |  |
| RBWH-HF-38 | 75 | Male | No |  |  | No | Yes | Yes | Yes | No | Yes | No | No | No | No | No | 2 | 72 | 100 | 65 |  | No | 15-25% | 131 | 7.7 | 3.26 | 2.6 | 138 | 3.9 | 114 | 12.7 | 70 | Yes | Yes | Yes | Yes | No | Yes | 01.11.2017 | No |  |
| RBWH-HF-39 | 61 | Female | No |  |  | No | Yes | Yes | No | No | Yes | No | No | No | No | No | 2 | 110 | 105 | 122 |  | No | 16% | 133 | 6.6 | 3.83 | 2.01 | 139 | 3.8 | 73 | 5.8 | 77 | Yes | Yes | Yes | Yes | No | No | 01.11.2017 | No |  |
| RBWH-HF-40 | 48 | Male | No |  |  | No | No | No | No | No | No | No | No | No | No | No | 1 | 70 | 64 | 78 |  | No | 30-35% | 143 | 5.2 | 2.42 | 2.24 | 136 | 4 | 96 | 6.8 | 86 | Yes | Yes | Yes | Yes | No | Yes | 01.11.2017 | No |  |
| RBWH-HF-41 | 82 | Male | No |  |  | No | Yes | No | No | No | No | No | No | Yes | No | No | 2,3 | 80 | 120 | 70 |  | No | 32% | 119 | 5.2 | 3.1 | 1.36 | 138 | 4.3 | 139 | 12.9 | 43 | Yes | Yes | No | Yes | No | No | 02.11.2017 | No |  |
| RBWH-HF-42 | 81 | Male | No |  |  | No | Yes | Yes | No | No | Yes | Yes | No | No | Yes | No | 2 | 64 | 105 | 70 | LBBB | Yes | 24% | 112 | 7.6 | 5.09 | 1.4 | 133 | 4.1 | 90 | 5.4 | 68 | No | Yes | Yes | Yes | Yes | No | 09.11.2017 | No |  |
| RBWH-HF-43 | 67 | Female | No |  |  | No | Yes | Yes | No | Yes | Yes | No | No | Yes | No | No | 3 | 90 | 120 | 60 |  | No | 29% | 103 | 6.1 | 3.57 | 1.61 | 141 | 4.6 | 98 | 8.3 | 59 | No | Yes | Yes | Yes | No | No | 17.11.2017 | CV hospitalisation (28.03.2018) |  |
| RBWH-HF-44 | 55 | Female | No |  |  | No | Yes | Yes | No | Yes | No | No | No | No | Yes | Yes | 3 | 88 | 130 | 72 |  | Yes | 25% | 127 | 9.9 | 6.04 | 2.67 | 140 | 4.2 | 111 | 18.7 | 49 | No | Yes | No | Yes | No | Yes | 20.11.2017 | CV hospitalisation (19.02.2018) |  |
| RBWH-HF-45 | 57 | Female | No |  |  | No | No | No | No | No | No | Yes | No | No | Yes | No | 3 | 110 | 90 | 55 |  | Yes | 10% | 101 | 6.6 | 4.32 | 0.97 | 135 | 4.5 | 84 | 5.2 | 64 | Yes | Yes | Yes | No | Yes | Yes | 11.12.2017 | No |  |
| RBWH-HF-46 | 61 | Male |  | Yes |  | No | Yes | Yes | Yes | Yes | No | No | No | No | No | No | 2 | 78 | 105 | 68 |  | No | 30% | 189 | 11.5 | 7.41 | 2.82 | 136 | 4.4 | 128 | 13.1 | 52 | Yes | Yes | Yes | No | No | No | 08.01.2018 | No |  |
| RBWH-HF-47 | 73 | Male |  | Yes |  | Yes | Yes | Yes | No | Yes | Yes | Yes | No | Yes | Yes | No | 2,3 | 80 | 150 | 60 | LBBB | Yes | 26% | 119 | 9.3 | 5.49 | 2.45 | 134 | 4.1 | 94 | 7.1 | 69 | Yes | No | No | No | Yes | Yes | 10.01.2018 | No |  |
| RBWH-HF-48 | 70 | Male | No |  |  | Yes | Yes | Yes | No | No | Yes | No | No | Yes | No | No | 2,3 | 76 | 120 | 74 |  | No | 25% | 115 | 7.6 | 4.56 | 1.96 | 137 | 4.1 | 84 | 4.6 | 81 | No | Yes | Yes | No | No | No | 17.01.2018 | No |  |
| RBWH-HF-49 | 70 | Male |  |  | Yes | No | Yes | No | No | Yes | Yes | No | No | Yes | No | No | 2,3 | 72 | 160 | 75 |  | No | 25% | 126 | 8.7 | 6.06 | 1.49 | 140 | 4.9 | 474 | 30.2 | 10 | No | Yes | No | Yes | No | No | 24.01.2018 | No |  |
| RBWH-HF-50 | 88 | Female | No |  |  | NA | Yes | Yes | No | Yes | Yes | No | No | No | No | Yes | 1 | 74 | 100 | 60 | NA | Yes | 20-25% | 141 | 14.2 | 10.2 | 2.19 | 138 | 3.8 | 77 | 7.1 | 58 | Yes | Yes | No | Yes | Yes | No | 25.01.2018 | CV hospitalisation (01.04.2018) |  |
| RBWH-HF-51 | 67 | Male | No |  |  | No | Yes | Yes | No | Yes | No | No | No | Yes | No | No | 2 | 80 | 122 | 70 |  | No | 35-40% | 134 | 6.6 | 2.99 | 2.9 | 138 | 4 | 65 | 62 | >90 | Yes | Yes | Yes | No | No | Yes | 29.01.2018 | No |  |
| RBWH-HF-52 | 72 | Male | No |  |  | No | Yes | Yes | No | Yes | No | No | No | No | No | No | 2,3 | 90 | 130 | 80 |  | Yes | 20% | 123 | 9.6 | 7.22 | 1.33 | 124 | 5.2 | 143 | 15 | 42 | Yes | Yes | No | No | No | No | 02.02.2018 | No |  |
| RBWH-HF-54 | 74 | Female | No |  |  | No | Yes | Yes | Yes | Yes | Yes | No | Yes | No | No | No | 2 | 88 | 118 | 70 |  | Yes | 32% | 104 | 6.3 | 6.3 | 0.97 | 135 | 3.8 | 96 | 6.9 | 55 | Yes | Yes | No | Yes | No | No | 06.02.2018 | No |  |
| RBWH-HF-55 | 67 | Male |  | Yes |  | No | Yes | Yes | No | No | Yes | Yes | No | No | No | No | 2,3 | 66 | 105 | 65 |  | No | 26% | 137 | 6.4 | 4.36 | 1.34 | 135 | 4 | 77 | 6.8 | 89 | Yes | Yes | Yes | No | No | No | 08.02.2018 | No |  |
| RBWH-HF-56 | 85 | Male |  |  | Yes | No | Yes | Yes | No | Yes | Yes | No | No | No | No | No | 2 | 70 | 120 | 72 |  | No | 38% | 115 | 6.8 | 5.42 | 0.6 | 139 | 4.2 | 119 | 13.8 | 48 | No | Yes | No | Yes | No | No | 08.02.2018 | No |  |
| RBWH-HF-57 | 53 | Female | No |  |  | No | No | No | No | No | Yes | No | No | No | No | No | 2 | 82 | 130 | 68 |  | No | 26% | 142 | 7.4 | 4.46 | 1.89 | 137 | 4.3 | 87 | 4.4 | >90 | Yes | Yes | Yes | No | No | No | 08.02.2018 | No |  |
| RBWH-HF-58 | 41 | Male | Yes |  |  | Yes | Yes | No | No | No | No | No | No | Yes | No | No | 2,3 | 80 | 100 | 60 |  | Yes | 16-20% | 138 | 6.2 | 3.92 | 1.67 | 137 | 3.6 | 137 | 4.5 | >90 | Yes | Yes | Yes | Yes | No | No | 08.02.2018 | No |  |
| RBWH-HF-59 | 57 | Male |  | Yes |  | No | Yes | No | No | No | No | No | No | No | No | Yes | 2 | 110 | 95 | 62 |  | Yes | 25% | 133 | 9.8 | 6.8 | 1.72 | 138 | 3.9 | 164 | 10.4 | 39 | Yes | Yes | No | Yes | No | No | 13.02.2018 | No |  |
| RBWH-HF-60 | 74 | Male | No |  |  | No | Yes | No | No | Yes | No | No | No | Yes | No | Yes | 2,3 | NA | NA | NA |  | No | 25-30% | 88 | 10 | 8.3 | 0.49 | 132 | 4.7 | 593 | 16.1 | 12 | Yes | Yes | No | Yes | No | Yes | 14.02.2018 | No |  |
| RBWH-HF-61 | 65 | Female |  | YES |  | No | No | Yes | No | Yes | No | Yes | No | No | Yes | No | 2,3 | NA | NA | NA |  | No | 25% | 93 | 8.2 | 6.55 | 0.76 | 133 | 3.3 | 223 | 42.1 | 19 | Yes | Yes | No | Yes | No | No | 16.02.2018 | CV hospitalisation(10.03.2018) |  |
| RBWH-HF-63 | 44 | Male | No |  |  | Yes | No | Yes | No | No | No | No | No | No | No | No | 2 | 150 | 110 | 72 |  | Yes | 30-35% | 169 | 7.3 | 5.22 | 1.28 | 135 | 5 | 111 | 9.9 | 74 | Yes | Yes | Yes | No | No | No | 22.02.2018 | No |  |
| RBWH-HF-64 | 67 | Female | No |  |  | No | No | Yes | No | No | Yes | No | No | Yes | No | No | 2,3 | NA | NA | NA |  | No | 15% | 104 | 7.3 | 4.52 | 1.82 | 137 | 4.4 | 65 | 6 | 79 | Yes | Yes | Yes | Yes | No | No | 01.03.2018 | No |  |
| RBWH-HF-65 | 33 | Male | No |  |  | No | No | No | No | No | No | No | No | No | No | No | 3 | NA | NA | NA |  | No | 15-20% | 166 | 11.9 | 7.15 | 2.89 | 139 | 5.4 | 138 | 10.3 | 65 | Yes | Yes | No | Yes | No | No | 10.04.2018 | No |  |
| RBWH-HF-66 | 64 | Male | Yes |  |  | No | Yes | No | No | Yes | Yes | No | No | No | No | No | 2 | NA | NA | NA |  | No | 30-35% | 125 | 11.4 | 8.57 | 1.34 | 126 | 4.3 | 108 | 15.1 | 61 | NA | NA | NA | NA | NA | NA | 11.04.2018 | CV hospitalisation(05.05.2018) |  |
| RBWH-HF-67 | 41 | Female | No |  |  | No | No | No | No | Yes | Yes | No | No | No | No | No | 2,3 | NA | NA | NA |  | No | 25-30% | 111 | 12.6 | 6.53 | 4.65 | 135 | 5.4 | 144 | 20.9 | 39 | Yes | Yes | No | Yes | No | No | 18.04.2018 | CV hospitalisation(25.05.2018) |  |
| RBWH-HF-68 | 84 | Female | No |  |  | No | No | No | No | No | No | No | No | No | Yes | No | 2,3 | NA | NA | NA |  | Yes | 25-30% | 158 | 7.7 | 5.37 | 1.59 | 136 | NA | 159 | 13.7 | 26 | Yes | Yes | No | No | Yes | No | 18.04.2018 | No |  |
| RBWH-HF-69 | 77 | Male | No |  |  | No | Yes | No | No | No | Yes | Yes | No | No | No | No | 3 | NA | NA | NA |  | No | 14% | 123 | 6.2 | 4.5 | 0.7 | 137 | 4.4 | 111 | 6.8 | 55 | No | Yes | No | Yes | No | No | 24.04.2018 | No |  |
| RBWH-HF-70 | 66 | Male |  | Yes |  | No | No | No | No | Yes | Yes | No | No | Yes | No | Yes | 3 | 102 | 80 | 65 |  | No | 10% | 117 | 7.1 | 4.09 | 1.75 | 134 | 4.3 | 226 | 19.5 | 23 | No | Yes | No | Yes | Yes | No | 26/04/2018 | CV hospitalisation(06.08.2018) |  |
| RBWH-HF-71 | 63 | Male | No |  |  | No | No | Yes | No | No | No | No | No | No | No | Yes | 2,3 | 88 | 100 | 68 |  | No | 30% | 126 | 6.1 | 4.96 | 0.73 | 130 | 5.4 | 824 | 19.4 | 7 | Yes | Yes | No | Yes | Yes | No | 9/05/2018 | CV hospitalisation(07.05.2018) | CV death (20.08.2018) |
| RBWH-HF-73 | 76 | Female | No |  |  | No | No | Yes | No | Yes | No | No | No | Yes | No | No | 2 | 90 | 130 | 70 |  | No | 37% | 91 | 9.5 | 7.03 | 1.16 | 135 | 4 | 279 | 31.5 | 14 | No | Yes | No | Yes | No | No | 13/06/2018 | No |  |
| RBWH-HF-74 | 71 | Female | No |  |  | No | No | Yes | Yes | Yes | No | No | No | No | No | Yes | 2 | 82 | 112 | 68 |  | No | 35% | 115 | 7.7 | 5.34 | 1.18 | 138 | 4.9 | 156 | 16.2 | 29 | No | Yes | No | Yes | No | No | 10/07/2018 | CV hospitalisation (12.02.2020) |  |
| RBWH-HF-75 | 75 | Male | No |  |  | No | No | No | No | Yes | Yes | No | No | No | No | Yes | 2 | 74 | 130 | 80 |  | No | 30-35% | 94 | 6.7 | 4.29 | 1.19 | 133 | 4.6 | 166 | 27.4 | 34 | No | No | Yes | No | No | No | 10/07/2018 | No |  |
| RBWH-HF-76 | 45 | Male | No |  |  | Yes | No | No | No | No | No | No | No | No | No | No | 2 | 90 | 110 | 70 |  | Yes | 25% | 172 | 8.4 | 5.09 | 2.53 | 139 | 4.2 | 91 | 4.5 | 88 | Yes | Yes | No | No | No | No | 18/07/2018 | No |  |
| RBWH-HF-77 | 87 | Male | No |  |  | No | Yes | No | No | No | No | No | Yes | Yes | No | Yes | 3 | 68 | 118 | 66 |  | No | 35-40 | 153 | 7.9 | 4.87 | 2.43 | 146 | 3.8 | 178 | 16.1 | 29 | Yes | Yes | No | Yes | Yes | No | 24/07/2018 | CV hospitalisation (15.06.2020) |  |
| RBWH-HF-78 | 60 | Male | No |  |  | No | No | Yes | No | No | No | No | No | Yes | No | Yes | 3 | 70 | 90 | 60 |  | No | 12% | 114 | 13 | 9.97 | 1.78 | 136 | 4.3 | 92 | 10.7 | 78 | Yes | Yes | Yes | Yes | No | No | 24/07/2018 | No |  |
| TPCH-HF-1 | 41 | Male | No |  |  | Yes | No | No | No | No | No | No | No | Yes | No | No | 2,3 | 80 | 100 | 68 | 98 | No | 12% | 155 | 6.4 | 2.88 | 2.4 | 136 | 3.8 | 99 | 5.9 | 81 | Yes | Yes | Yes | No | No | No | 09.05.2017 | No |  |
| TPCH-HF-10 | 67 | Male |  | Yes |  | No | No | No | No | No | Yes | No | No | No | No | No | 2 | 54 | 90 | 60 |  |  | 30-35% | 142 | 4.3 | 2.28 | 1.65 | 137 | 3.5 | 92 | 7.6 | 75 | Yes | Yes | No | No | No | No | 22.09.2017 | No |  |
| TPCH-HF-11 | 43 | Male | No |  |  | No | No | No | No | No | No | No | No | No | No | No | 2,3 | 60 | 87 | 55 |  | No | 27% | 138 | 4.8 | 2.87 | 1.44 | 138 | 4.2 | 104 | 6.9 | 76 | Yes | Yes | Yes | No | No |  | 24.10.2017 | No |  |
| TPCH-HF-12 | 58 | Female |  |  | Yes | No | Yes | No | No | No | Yes | No | No | No | Yes | No | 2 | 84 | 136 | 94 |  | Yes | 31% | 149 | 5.8 | 3.44 | 1.58 | 137 | 3.7 | 71 | 3.3 | 81 | Yes | Yes | No | No | Yes | No | 24.10.2017 | No |  |
| TPCH-HF-13 | 38 | Male | No |  |  | No | No | No | No | No | No | No | Yes | No | No | Yes | 3 | 80 | 84 | 58 | 133 | No | 6% | 154 | 5.8 | 3.39 | 1.42 | 138 | 4.5 | 103 | 4.8 | 79 | Yes | Yes | Yes | No | No | No | 17.11.2017 | No |  |
| TPCH-HF-14 | 62 | Male |  | Yes |  | No | Yes | Yes | No | No | No | No | No | No | Yes | No | 3 | 90 | 122 | 80 | 104 | No | 30% | 181 | 9.3 | 5.66 | 2.62 | 134 | 3.9 | 83 | 6.3 | 87 | Yes | No | No | Yes | No | No | 01.12.2017 | No |  |
| TPCH-HF-15 | 62 | Male |  | Yes |  | No | No | No | Yes | No | Yes | No | No | Yes | No | No | 2,3 | 96 | 118 | 65 | 183 | No | 20% | 144 | 4.6 | 3.2 | 0.7 | 136 | 3.8 | 67 | 4.1 | >90 | No | No | Yes | Yes | No | No | 16.02.2018 | No |  |
| TPCH-HF-16 | 43 | Male | No |  |  | No | No | No | No | No | Yes | No | No | Yes | Yes | Yes | 2,3 | 116 | 90 | 62 |  | No | 20% | 177 | 5.9 | 3.92 | 1.39 | 138 | 4.4 | 102 | 6.3 | 77 | Yes | Yes | Yes | No | No | Yes | 16.02.2018 | CV death (18.04.2018) |  |
| TPCH-HF-17 | 84 | Male | No |  |  | No | No | No | No | No | Yes | No | No | No | NA | Yes | 2,3 | NA | NA | NA |  | No | 19% | 129 | 5.6 | 3.5 | 1.28 | 138 | 4.4 | 102 | 4.9 | 62 | Yes | Yes | Yes | No | No | No | 21.02.2018 | No |  |
| TPCH-HF-2 | 40 | Male | No |  |  | NA | No | No | Yes | No | No | No | No | Yes | No | Yes | 2,3 | 96 | 100 | 62 | 92 | No | 15% | 140 | 8 | 4.77 | 2.54 | 139 | 4.5 | 123 | 6.2 | 63 | Yes | No | Yes | No | Yes |  | 07.07.2017 | No |  |
| TPCH-HF-3 | 47 | Male | No |  |  | NA | No | No | Yes | No | No | No | No | Yes |  | NA | 2,3 | 72 | 94 | 62 | 182 | Yes | 15% | 138 | 6.1 | 2.7 | 2.36 | 138 | 3.6 | 80 | 9.6 | >90 | Yes | Yes | Yes | No | No | Yes | 30.06.2017 | CV hospitalisation (16.11.2017) |  |
| TPCH-HF-4 | 60 | Male |  | Yes |  | Yes | No | No | No | No | No | No | No | No | Yes | No | 2,3 | 96 | 110 | 76 | 124 | No | 11% | 144 | 9.3 | 5.56 | 2.67 | 138 | 4.2 | 132 | 80 | 61 | Yes | No | Yes | No | No | Yes | 07.07.2017 | No |  |
| TPCH-HF-5 | 48 | Male |  |  | Yes | No | No | No | No | No | No | No | No | Yes | No | No | 2 | 71 | 102 | 70 | 230 | No | 30-35% | 161 | 9.2 | 6.47 | 1.69 | 129 | 4.1 | 153 | 14 | 46 | Yes | No | Yes | No | No | Yes | 11.08.2017 | CV Hospitalisation (23.10.2017) | CV death (09.01.2018) |
| TPCH-HF-6 | 71 | Female | No |  |  | No | No | Yes | No | No | No | No | No | No | No | No | 2 | 72 | 118 | 60 |  | No | 38% | 116 | 6 | 2.71 | 2.44 | 138 | 4.4 | 221 | 15.4 | 19 | Yes | Yes | No | No | No | No | 11.08.2017 | CV hospitalisation (11.06.2019) |  |
| TPCH-HF-7 | 70 | Male | No |  |  | No | No | Yes | Yes | No | Yes | No | No | No | No | No | 2,3 | 66 | 100 | 64 |  | No | 19% | 120 | 7.9 | 5.42 | 1.81 | 130 | 5 | 154 | 19.8 | 39 | Yes | Yes | Yes | No | Yes | Yes | 22.09.2017 | CV hospitalisation (29.08.2019) |  |
| TPCH-HF-8 | 59 | Female | No |  |  | No | No | No |  | No | No | No | No | Yes | No | Yes | 2,3 | 60 | 95 | 50 |  | No | 11% | 143 | 4.9 | 3.39 | 0.9 | 134 | 5 | 125 | 30.9 | 41 | Yes | Yes | Yes | No | No | Yes | 22.09.2017 | CV hospitalisation (24.10.2017) |  |
| TPCH-HF-9 | 23 | Male |  | Yes |  | No | No | No | No | No | No | No | No | Yes | No | Yes | 2,3 | 80 | 90 | 60 | 108 | No | 18% | 132 | 6.2 | 3.64 | 1.44 | 132 | 4.6 | 79 | 5.4 | >90 | Yes | Yes | Yes | No | Yes | Yes | 22.09.2017 | CV hospitalisation (28.09.2019) |  |

| Demographic History | Mean Age | 64.8 |
| --- | --- | --- |
|  | Sex (male:female) | 67:31 |
|  | Current smoker | 7 |
|  | Exsmoker (Yes) | 18 |
|  | Never Smoked | 76 |
|  | Alcohol intake (Current) | 10 |
| Etiology | Hypertention | 50 |
|  | Ischemic heart disease (IHD) | 49 |
|  | Valuar heart disease (VHD) | 11 |
| Other Mediacl coditions | Diabetes mellitus | 33 |
|  | Dyslipidemia | 50 |
|  | Chronic Obstructive Pulmonary Disease (COPD) | 15 |
|  | Stroke | 6 |
|  | Past medical history of HF | 45 |
|  | Pleural effusion | 16 |
|  | Peripheral oedema | 19 |
| Vital Signs | Heart rate | Average 78.8 |
|  | Systolic blood pressure (mmHg) | Average 112.2 |
|  | Diastolic blood pressure (mmHg) | Average 68.6 |
| Treatments | ACEI or ARB | 67 |
|  | Beta-blocker | 87 |
|  | Spironolatone/Eplerenone | 54 |
|  | Loop Diuretic | 55 |
|  | Digoxin | 19 |
|  | Intra cardiac device (ICD) | 26 |
| Events (cardiovascular death or rehospitalisation due to HF) | | 29 |
